# IL-2 Suppression of IL-12p70 by a Recombinant HSV-1 Expressing IL-2 Induces T-Cell Auto-Reactivity and CNS Demyelination

**DOI:** 10.1371/journal.pone.0016820

**Published:** 2011-02-18

**Authors:** Mandana Zandian, Kevin R. Mott, Sariah J. Allen, Shuang Chen, Moshe Arditi, Homayon Ghiasi

**Affiliations:** 1 Department of Surgery, Center for Neurobiology and Vaccine Development, Ophthalmology Research, Cedars-Sinai Medical Center, Los Angeles, California, United States of America; 2 Division of Pediatric Infectious Diseases and Immunology, Cedars-Sinai Medical Center, Los Angeles, California, United States of America; 3 Department of Medicine, David Geffen School of Medicine at UCLA, Los Angeles, California, United States of America; University of California, Los Angeles, and Cedars-Sinai Medical Center, United States of America

## Abstract

To evaluate the role of cellular infiltrates in CNS demyelination in immunocompetent mice, we have used a model of multiple sclerosis (MS) in which different strains of mice are infected with a recombinant HSV-1 expressing IL-2. Histologic examination of the mice infected with HSV-IL-2 demonstrates that natural killer cells, dendritic cells, B cells, and CD25 (IL-2rα) do not play any role in the HSV-IL-2-induced demyelination. T cell depletion, T cell knockout and T cell adoptive transfer experiments suggest that both CD8^+^ and CD4^+^ T cells contribute to HSV-IL-2-induced CNS demyelination with CD8^+^ T cells being the primary inducers. In the adoptive transfer studies, all of the transferred T cells irrespective of their CD25 status at the time of transfer were positive for expression of FoxP3 and depletion of FoxP3 blocked CNS demyelination by HSV-IL-2. The expression levels of IL-12p35 relative to IL-12p40 differed in BM-derived macrophages infected with HSV-IL-2 from those infected with wild-type HSV-1. HSV-IL-2-induced demyelination was blocked by injecting HSV-IL-2-infected mice with IL-12p70 DNA. This study demonstrates that suppression of the IL-12p70 function of macrophages by IL-2 causes T cells to become auto-aggressive. Interruption of this immunoregulatory axis results in demyelination of the optic nerve, the spinal cord and the brain by autoreactive T cells in the HSV-IL-2 mouse model of MS.

## Introduction

Epidemiologic studies have implicated genetic, as well as environmental factors, in the development of multiple sclerosis (MS) [1], [2]. The possibility that infectious agents, particularly viruses, are involved [3], [4] remains controversial [5], [6], [7] and the evidence suggests that if an infectious agent is involved, it alone may not be sufficient to initiate the observed pathology [5], [6], [7]. There are several lines of evidence implicating the cytokine, interleukin-2 (IL-2) in the pathology of MS [8], [9], [10], [11]. Patients with MS have elevated levels of IL-2 in their cerebrospinal fluid (CSF) and sera and IL-2-deficient mice are more resistant to experimental autoimmune encephalitis (EAE) than their heterozygote and wild-type counterparts [12]. To explore the possibility that IL-2 may play a role in the pathology of MS in conjunction with viral infection, we constructed a recombinant herpes simplex virus type 1 (HSV-1) that expresses murine IL-2 constitutively [13] as well as a panel of control recombinant viruses that express murine IL-4, interferon (IFN)-γ, IL-12p35, or IL-12p40 continuously [14], [15], [16]. We have shown previously that ocular infection of different strains of mice (*i.e.*, BALB/c, C57BL/6, SJL/6, and 129SVE) with the HSV-IL-2 virus results in demyelination of the optic nerves (ON), the spinal cords (SC) and the brains of the infected mice as determined by histologic examination of tissues obtained at necropsy [17], [18]. The demyelinated lesions involved the periventricular white matter, brain stem, and SC white matter, and had striking similarities to the plaques seen in patients with MS [17]. Demyelination was detected in the CNS of infected mice up to 75 days (the longest time point tested) post HSV-IL-2 infection. However, the severity of demyelination did not increase from 14 days to 75 days post infection. In addition, the HSV-IL-2 infected mice developed optic neuropathy as determined by changes in the visual-evoked cortical potentials (VECPs) [18]. In contrast, demyelination was not detectable after infection of the mice with wild-type (wt) HSV-1 alone or infection of the mice with the HSV-IL-4 or HSV-IFN-γ viruses, which are identical to HSV-IL-2 except that they express IL-4 or IFN-γ instead of IL-2 [17], [18].

The pathogenic processes that underlie demyelination in MS have not yet been elucidated. One hypothesis is that autoimmunity to CNS antigens is triggered by environmental factors in genetically susceptible individuals and that the activated immune response leads to destruction of the myelin [1], [2]. Our published studies suggest that HSV plays an important role in initiating destruction of the myelin in the presence of elevated levels of IL-2. Thus, the HSV-IL-2-induced demyelination could be associated with the innate or the adaptive arms of the immune response in the infected mice. Therefore, we undertook the current studies to determine the role of NK-cells, dendritic cells (DCs), B-cells, IL-2rα (CD25), CD4^+^ T cells, and CD8^+^ T cells in the HSV-IL2-induced CNS demyelination. The results indicate that the demyelinated lesions observed after infection with HSV-IL-2 are associated with both CD4^+^ and CD8^+^ T cells, with CD8^+^ T cells playing the more prominent role. Furthermore, the demyelination was found to be associated with IL-2-mediated suppression of IL-12p70 expression by macrophages. The expression of IL-12p70 in the macrophages in the presence of IL-2 was restored by co-infection of mice with recombinant HSV-IL-12p70 virus, which also prevented demyelination. Thus, IL-12p70 expression by macrophages can act to regulate induction of T-cell autoimmune disease. Our results suggest a potential mechanism for the association of higher levels of IL-2 expression with susceptibility to MS, in which the IL-2 inhibits the ability of the macrophages to suppress an autoimmune T-cell response to infection with neurotropic viruses.

## Results

### Role of B cells, DCs, and NK cells in HSV-IL-2 induced demyelination in infected mice

**Figure 1:**
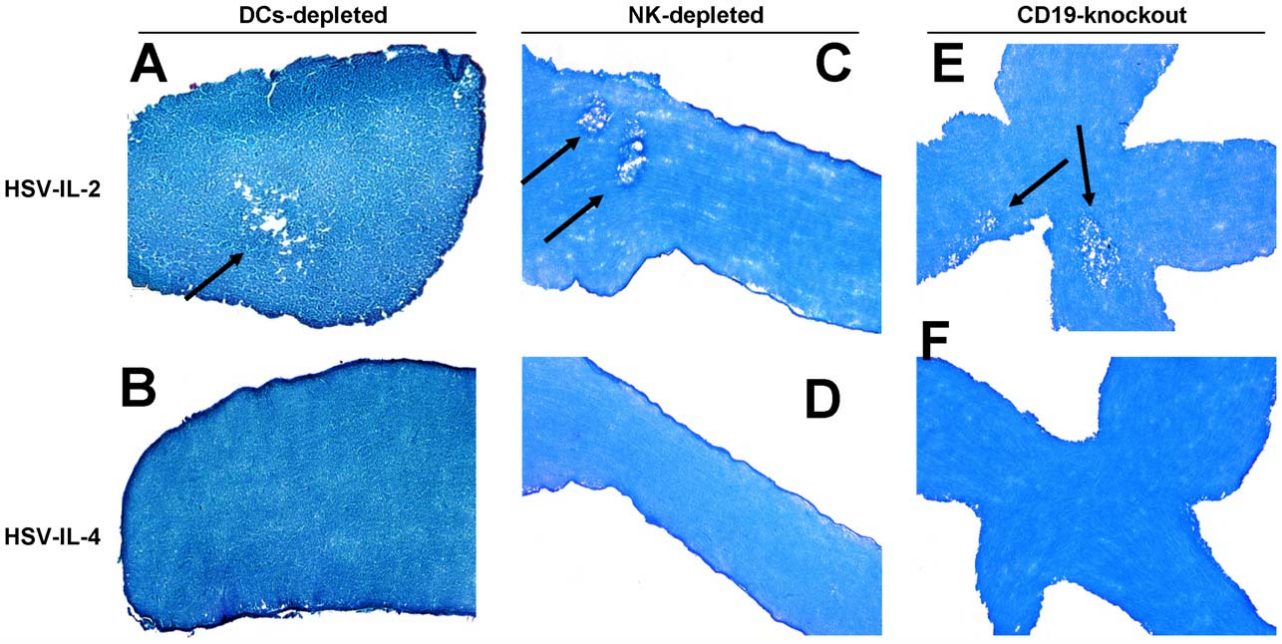
Role of innate immunity in HSV-IL-2 induced CNS demyelination. Female BALB/c-CD19^−/−^, BALB/c-DTR transgenic mice, and NK-depleted C57BL/6 mice were infected ocularly with HSV-IL-2 or HSV-IL-4 as described in Materials and Methods. On day 14 PI, ON were collected, fixed, sectioned, and stained with LFB. Representative photomicrographs are shown. Arrows indicate areas of demyelination.

To determine the possible involvement of innate immunity in the regulation of HSV-IL-2-induced demyelination in the present study, we used B-cell deficient mice (BALB/c-CD19^−/−^), BALB/c-DTR transgenic mice (CD11c-diphtheria toxin receptor-GFP) that were depleted of DCs using DT, and C57BL/6 mice that were depleted of NK cells using anti-asialo GM1 monoclonal antibodies. All of the mice, including the female BALB/c-CD19^−/−^, control BALB/c, BALB/c-DTR transgenic mice with and without DC depletion, and C57BL/6 mice with and without NK depletion, were infected ocularly with either HSV-IL-2 or HSV-IL-4. At day 14 PI, the mice were sacrificed and the ONs collected post-fixed and stained with the myelin stain, LFB. Representative photomicrographs of the ON sections from mice infected with HSV-IL-2 or HSV-IL-4 are shown in Figure 1.

Demyelination was observed in the HSV-IL-2-infected BALB/c-DTR mice that had been depleted of their DCs (Fig. 1A) but not in the HSV-IL-4-infected BALB/c-DTR mice that had been depleted of their DCs (Fig. 1B). Similarly, demyelination of the ON was observed in HSV-IL-2-infected mice that had been depleted of their NK cells (Fig. 1C) but not in NK-depleted mice following ocular infection with HSV-IL-4 (Fig. 1D).

Finally, demyelination of the ON was observed in the HSV-IL-2-infected BALB/c-CD19^−/−^ mice (Fig. 1E), but not in the HSV-IL-4-infected BALB/c-CD19^−/−^ mice (Fig. 1F). The patterns of demyelination of the ON in the absence of DCs, NK cells, or B cells were similar to the patterns of demyelination observed in wt mice infected with HSV-IL-2 (data not shown). Similar results were obtained with regards to demyelination in the SC and brains of the infected mice (data not shown). The failure of depletion of the cells to block the HSV-IL-2-induced demyelination suggests that B cells, NK cells, and DCs do not contribute to the demyelination of the ON in this mouse model. As the depletion did not result in exacerbation of the demyelination, the results also suggest that these components of the innate immune response do not act to suppress the autoimmune response. We have observed that the absence of macrophages exacerbates HSV-IL-2-induced demyelination and that even wt HSV-1 induces demyelination in infected BALB/c or C57BL/6 mice in the absence of macrophages (data not shown), which suggests that macrophages are not required for the development of the autoreactive demyelinating response, but may act to suppress it, as discussed in detail below.

### Role of T cells in HSV-IL-2-induced demyelination

**Figure 2:**
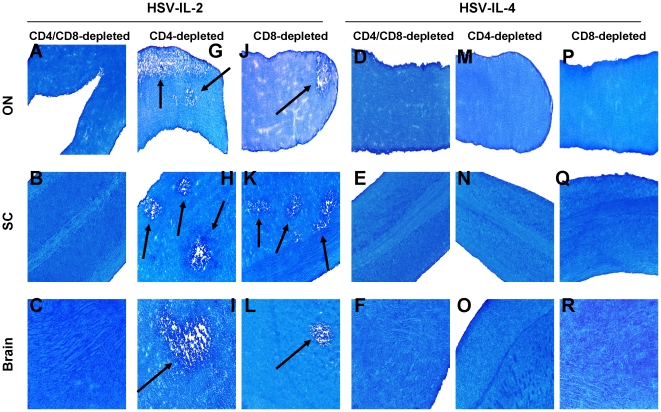
Demyelination in T cells-depleted mice. Mice were infected ocularly with HSV-IL-2 or HSV-IL-4. Forty-eight hr prior to infection, and on days 1, 5, and 9 days PI mice were depleted of CD4^+^ T cell, CD8^+^ T cell, or both T cell populations as described in Materials and Methods. After 14 days, brains, SC, and ON were removed, sectioned, and stained for LFB. Arrows indicate areas of demyelination in ON, SC, and brains of HSV-IL-2 infected mice.

To determine whether T cells contribute to the HSV-IL-2-induced demyelination, BALB/c mice were depleted of CD4**^+^** and CD8**^+^** cells and infected ocularly with HSV-IL-2 or control HSV-IL-4. Demyelination was not detectable in ON, brain, or SC of BALB/c mice depleted of both CD4**^+^** and CD8**^+^** T cells and infected ocularly with HSV-IL-2 (Fig. 2, Panels A, B, C). Similarly, demyelination was not detectable in C57BL/6 mice depleted of both CD4**^+^** and CD8**^+^** cells and infected ocularly with HSV-IL-2 (data not shown). As expected mice of either strain that were depleted of CD4^+^ and CD8^+^ T cells and infected with HSV-IL-4 rather than HSV-IL-2 showed no sign of demyelination in ON (Fig. 2, Panels D, E, and F). These results suggested that, as has been described in MS and other models of MS [19], [20], [21], T cells do contribute to the HSV-IL-2-induced demyelination.

**Figure 3:**
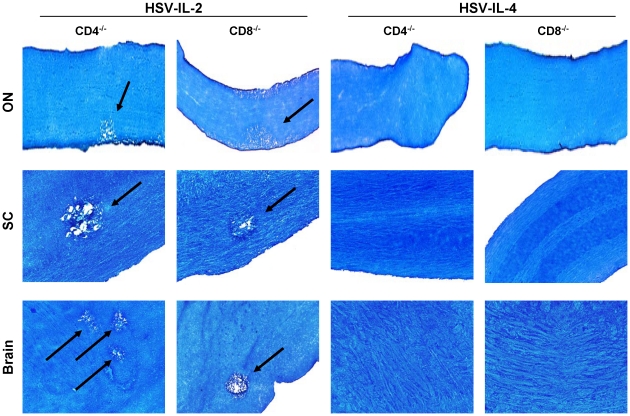
Demyelination in T cells-knockout mice. CD4^−/−^ and CD8^−/−^ mice were infected ocularly with HSV-IL-2 or HSV-IL-4 as described in Materials and Methods. After 14 days, ON, SC, and brains were removed, sectioned, and stained for LFB. Arrows indicate areas of demyelination in ON, SC, and brain of HSV-IL-2 infected mice.

HSV-IL-2-infection of the BALB/c mice that had been depleted of their CD4^+^ T cells, but not their CD8^+^ cells, resulted in demyelination in the ON, SC, and brain, which was detected as the pale blue area in LFB staining (Fig. 2, Arrows, Panels G, H, and I). Similarly, HSV-IL-2 infection of mice that had been depleted of their CD8^+^ T cells but not their CD4^+^ cells, resulted in demyelination in their ON, SC, and brain (Fig. 2, arrows; Panels J, K, and L). As expected neither CD4-depleted nor CD8-depleted mice infected with HSV-IL-4 showed any signs of demyelination (Fig. 2, Panels M-R). Similar results were obtained when C57BL/6 mice were depleted of their CD4**^+^** T cells alone or CD8**^+^** T cells alone and infected ocularly with HSV-IL-2 or control HSV-IL-4 (data not shown). As depletion of both CD4^+^ and CD8^+^ T cells prevented the HSV-IL-2-induced demyelination, these results suggest that both CD4^+^ and CD8^+^ T cells contribute to the CNS demyelination. To verify these results, we analyzed demyelination in CD4 (C57BL/6-CD4^−/−^) and CD8 (C57BL/6-CD8^−/−^) knockout mice. Demyelination was observed in the ON, SC, and brain of the CD4 knockout mice infected with HSV-IL-2 (Fig. 3, CD4^−/−^) and the ON, SC, and brain of the CD8 knockout mice infected with HSV-IL-2 (Fig. 3, CD8^−/−^). No demyelination was observed in ON, SC, and brain of CD4 knockout mice (Fig. 3, CD4^−/−^) or CD8 knockout mice infected with HSV-IL-4 (Fig. 3, CD4^−/−^). Collectively, these data strongly implicate both CD4^+^ and CD8^+^ T cells in HSV-IL-2-induced CNS demyelination.

We noted that the severity of the HSV-IL-2-induced demyelination appeared to be greater in the presence of CD8^+^ T cells (in the CD4 knockout mice) than in the presence of CD4^+^ T cells (in the CD8 knockout mice) (Fig. 3). Thus, to determine if more plaques are present in the CNS of CD4-knockout mice as compared with their CD8-knockout counterparts or wt C57BL/6 mice, we counted the number of observed plaques in the ON, SC and brain of CD4^−/−^, CD8^−/−^, and wt control mice. The data are shown as the number of sections showing demyelination plaques per total stained sections counted in Figure S1. More plaques were detected in the brains of both the HSV-IL-2-infected CD4- and CD8-knockout mice than in the brains of the HSV-IL-2-infected wt mice, but these differences were only significant when the numbers of plaques in the brains of the CD4-knockout mice were compared with the numbers in the brains of the wt mice (Figure S1-A; Brain; p<0.05). The number of HSV-IL-2-induced plaques detected in the SC were significantly higher in both the CD4- and CD8- knockout mice as compared with the numbers of plaques in the SC of wt mice (Figure S1-A, SC). In contrast, No significant differences were observed in the number of HSV-IL-2-induced plaques detected in ON of the knockout mice and the wt control mice (Figure S1-A, ON). Overall, more HSV-IL-2-induced demyelination plaques were detected in the SC of the knockout mice than their ON or brain (Figure S1-A). The number of plaques detected in brain, SC, and ON of depleted and mock-depleted BALB/c mice are presented in Figure 1B (Figure S1-B). Similar to knockout mice, more plaques were present in the CNS of CD4-depleted mice compare with their CD8-depleted counterparts but these differences did not reach statistical significance compared with the number of plaques in the mock-depleted control mice (Figure S1-B). As anticipated, the CD4-, CD8-, or mock-depleted groups of mice had significantly higher number of plaques than mice that were depleted of both CD4^+^ and CD8^+^ T cells (Figure S1-B) Thus, our CD4 and CD8 knockout studies suggest that both CD4 and CD8 T cells contribute to CNS demyelination and in the absence of the missing T cell subset the level of demyelination is increased in HSV-IL-2 infected mice.

### Role of natural and HSV-IL-2-induced T cells in CNS demyelination

**Figure 4:**
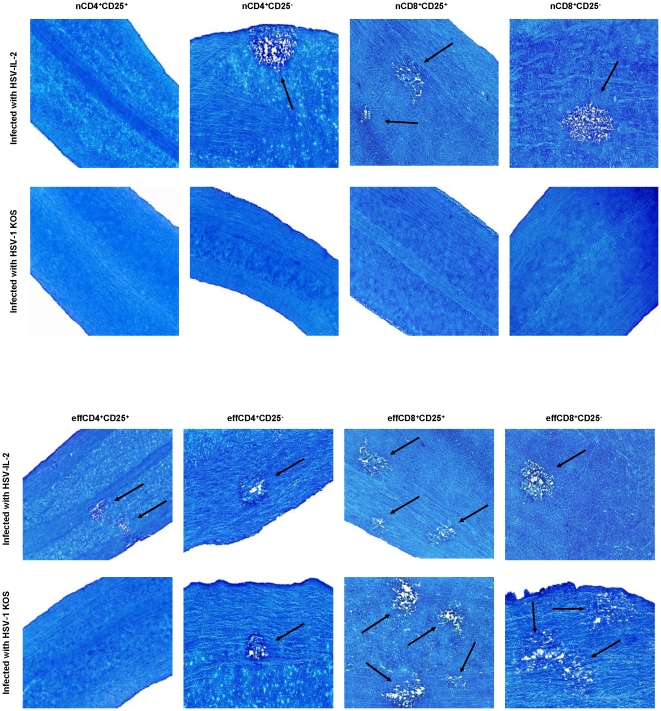
Adoptive transfer of naive and effector T cells to SCID mice. Naive T cells (nCD8^+^CD25^+^, nCD8^+^CD25^−^, nCD4^+^CD25^+^, and nCD4^+^CD25^−^) were isolated from naive mice, while the effector T cells (effCD8^+^CD25^+^, effCD8^+^CD25^−^, effCD4^+^CD25^+^, and effCD4^+^CD25^−^) were isolated from mice infected with HSV-IL-2 on day 5 PI. Magnetically isolated cells were transferred IP into recipient SCID mice and 4hr post-adoptive transfer, recipient SCID mice were infected ocularly with HSV-IL-2 or WT HSV-1 strain KOS. Representative ON sections on day 14 PI from infected mice are shown. Arrows indicate areas of demyelination.

As the above results suggested that not only are both CD4^+^ and CD8^+^ T cells are involved in CNS demyelination but that CD8^+^ T cells play a more prominent role (Figures 2, 3), we extended the studies to differentiate between responses of CD4^+^ or CD8^+^ naive T cells verses effectors T cells in CNS demyelination. CD4^+^CD25^+^, CD4^+^CD25^−^, CD8^+^CD25^+^, and CD8^+^CD25^−^ T cells were isolated using magnetic beads from naive BALB/c mice (naive T cell, nT cell) or BALB/c mice infected ocularly with HSV-IL-2 (effector T cell, effT cell) and the cells injected intraperitoneally into BALB/c-SCID recipient mice. Four hours after adoptive transfer, all of the SCID recipient mice were infected ocularly with HSV-IL-2 or HSV-1 strain KOS. Control SCID mice that received tissue culture media only also were infected ocularly with HSV-IL-2 or KOS. Fourteen days after infection, the mice were sacrificed and the SC removed, post-fixed and stained with LFB. Representative photomicrographs are shown in Figure 4 and a summary of the data concerning the demyelination of the SC sections from mice infected with HSV-IL-2 or HSV-1 KOS is shown in Table 1.

**Table 1:** Summary of LFB staining for presence of demyelination in ON, SC, and brain of SCID mice following adoptive transfer.^a^

	Number of mice with CNS demyelination following infection with^c^	
Adoptive transfer b	HSV-IL-2	WT KOS
nCD4^+^CD25^+^	0/5 (0%)	0/5 (0%)
nCD4^+^CD25^−^	5/5 (100%)	0/5 (0%)
nCD8^+^CD25^+^	5/5 (100%)	0/5 (0%)
nCD8^+^CD25^−^	5/5 (100%)	0/5 (0%)
effCD8^+^CD25^+^	5/5 (100%)	5/5 (100%)
effCD8^+^CD25^−^	5/5 (100%)	5/5 (100%)
effCD4^+^CD25^+^	5/5 (100%)	0/5 (0%)
effCD4^+^CD25^−^	5/5 (100%)	5/5 (100%)
Media	0/5 (0%)	0/5 (0%)

a Presence of demyelination in ON, SC, and brain of 5 recipient mice per group were assessed on day 14 PI.

b Naive T cells (nCD8^+^CD25^+^, nCD8^+^CD25^−^, nCD4^+^CD25^+^, and nCD4^+^CD25^−^) were isolated from naive mice, while the effector T cells (effCD8^+^CD25^+^, effCD8^+^CD25^−^, effCD4^+^CD25^+^, and effCD4^+^CD25^−^) were isolated from mice infected with HSV-IL-2 on day 5 PI. Each SCID mice received 2×10^5^ cells in 300 µl of tissue culture media or tissue culture media only.

c Differences between group with CNS demyelination versus group with no CNS demyelination were statistically significant (p = 0.008, n = 5, Fisher exact test).

We found that demyelination was undetectable in the mice that received nCD4^+^CD25^+^ T cells prior to infection with HSV-IL-2 (Fig. 4, HSV-IL-2), whereas demyelination occurred in the mice that received nCD4^+^CD25^−^ T cells prior to infection (Fig. 4, HSV-IL-2, arrow). Demyelination was detectable in mice that received either nCD8^+^CD25^+^ or nCD8^+^CD25^−^ prior to infection with HSV-IL-2 virus (Fig. 4, HSV-IL-2, arrows). There were no signs of demyelination in KOS-infected mice that received nCD4^+^CD25^+^, nCD4^+^CD25^−^, nCD8^+^CD25^+^, or nCD8^+^CD25^−^ T cells (Fig. 4, KOS).

In contrast, demyelination in the SC was observed in all SCID mice that received effCD4^+^CD25^+^, effCD4^+^CD25^−^, effCD8^+^CD25^+^, or effCD8^+^CD25^−^ T cells and were infected with HSV-IL-2 (Fig. 4, HSV-IL-2, arrows). Demyelination was not observed in mice that received effCD4^+^CD25^+^ T cells and were infected with KOS (Fig. 4, KOS), although mice that received effCD4^+^CD25^−^, effCD8^+^CD25^+^, or effCD8^+^CD25^−^ T cells and were infected with KOS developed demyelination (Fig. 4, KOS, arrows). As expected SCID mice that received induced T cells but were not subsequently infected did not show any sign of demyelination (data not shown). Furthermore, SCID mice that received media without T cells prior to infection with HSV-IL-2 or KOS did not develop any demyelination (Table 1). The patterns of demyelination in the brain and ON of the different groups of infected mice were similar to the patterns of demyelination in the SC described above (data not shown). The results of these studies provide further evidence that both CD4^+^ and CD8^+^ T cells contribute to CNS demyelination with CD8^+^ T cells playing a more prominent role than CD4^+^ T cells.

### FoxP3 expression in brain of T cells recipient mice

**Figure 5:**
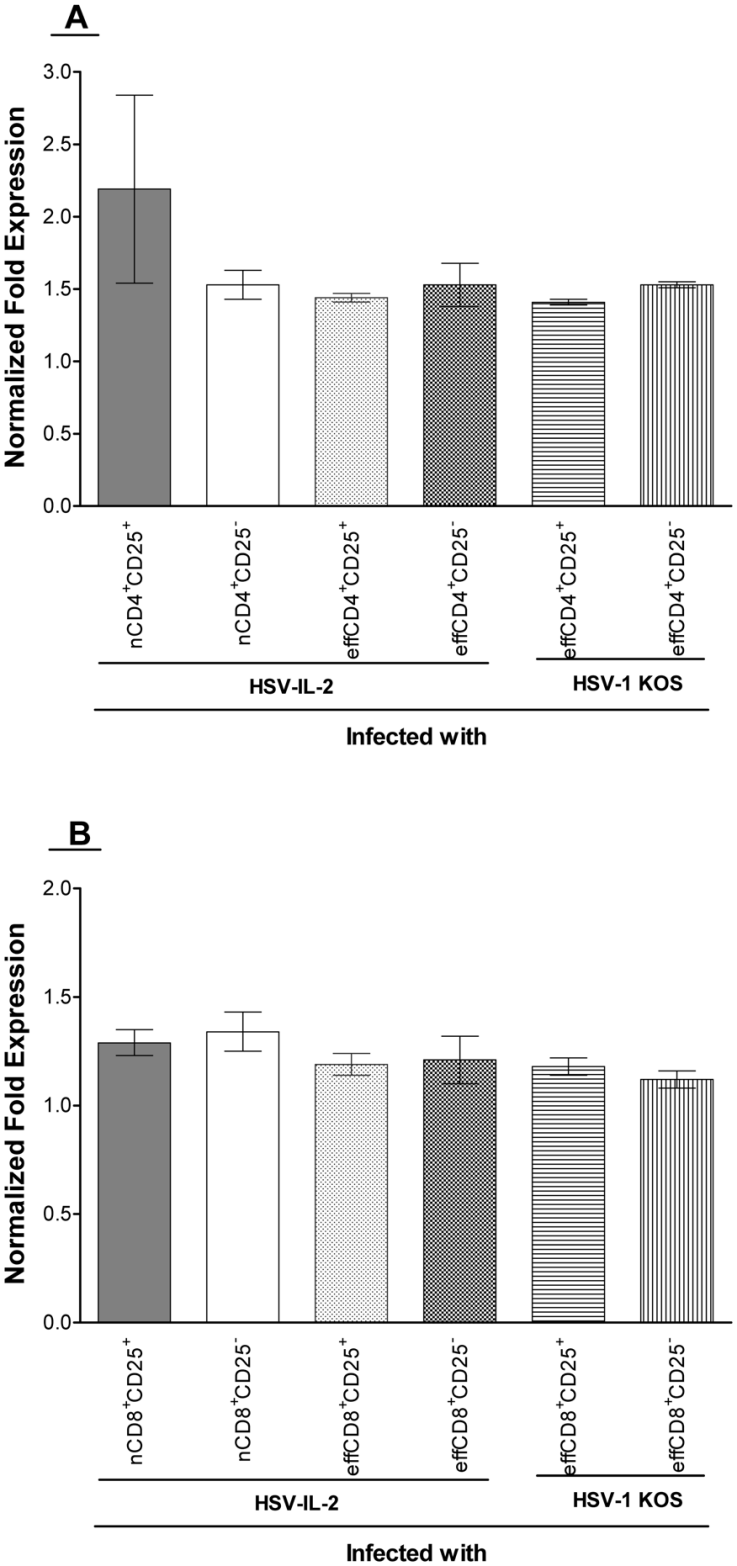
qRT-PCR analyses of FoxP3 transcript in brain of recipient SCID mice. Total RNA from brains of SCID mice described in Fig. 4 and Table 1 was isolated on day 14 PI. FoxP3 expression in SCID mice infected with the virus and without T cells transfer was used to estimate the relative expression of FoxP3 transcript in brains of each group of recipient SCID mice. GAPDH expression was used to normalize the relative expression of each transcript in brain of each group of mice. Each point represents the mean ± SEM from 3 mice. Panels: A) FoxP3 transcript isolated from SCID mice received CD4^+^ T cells; and B) FoxP3 transcript isolated from SCID mice received CD8^+^ T cells.

The results described above (Fig. 5) suggested that both CD25^+^ and CD25^−^ T cells can induce CNS demyelination in SCID mice that are infected with HSV-IL-2. To determine if the transferred T cells are expressing FoxP3, we measured FoxP3 transcripts by qRT-PCR using total RNA isolated from the brains of the mice described above and in Figure 5 that were sacrificed on day 14 PI. FoxP3 transcripts were found in the brains of the mice that received nCD4^+^CD25^+^, nCD4^+^CD25^−^, effCD4^+^CD25^+^, or effCD4^+^CD25^−^ cells and were infected with HSV-IL-2, and the mice that received effCD4^+^CD25^+^ or effCD4^+^CD25^−^ cells and were infected with KOS (Fig. 5A). The levels of the Foxp3 transcripts were similar in all the groups of mice irrespective of the CD25 status of the transferred cells (Fig. 5A). No differences in the expression of the CD4 transcript were observed among the groups (data not shown). Similarly, FoxP3 transcripts were observed in the brains of the mice that received nCD8^+^CD25^+^, nCD8^+^CD25^−^, effCD8^+^CD25^+^, or effCD8^+^CD25^−^ and were infected with HSV-IL-2, and the mice that received effCD8^+^CD25^+^ or effCD8^+^CD25^−^ and were infected with KOS (Fig. 5B). The expression of CD8 transcript was similar among these groups and statistically significant differences were not observed (data not shown). Similar level of FoxP3 transcript was detected in the isolated T cell population before transfer (not shown). Collectively, these results suggest that both the CD25^+^ and CD25^−^ T cells expressed FoxP3 transcripts and that the transferred T cells may have a Treg rather then a Teff phenotype. Previously it was reported that FoxP3 is a better indicator of Treg cell linage specification factor than CD25 [22], [23].

### Role of FoxP3 in HSV-IL-2-induced demyelination

**Figure 6:**
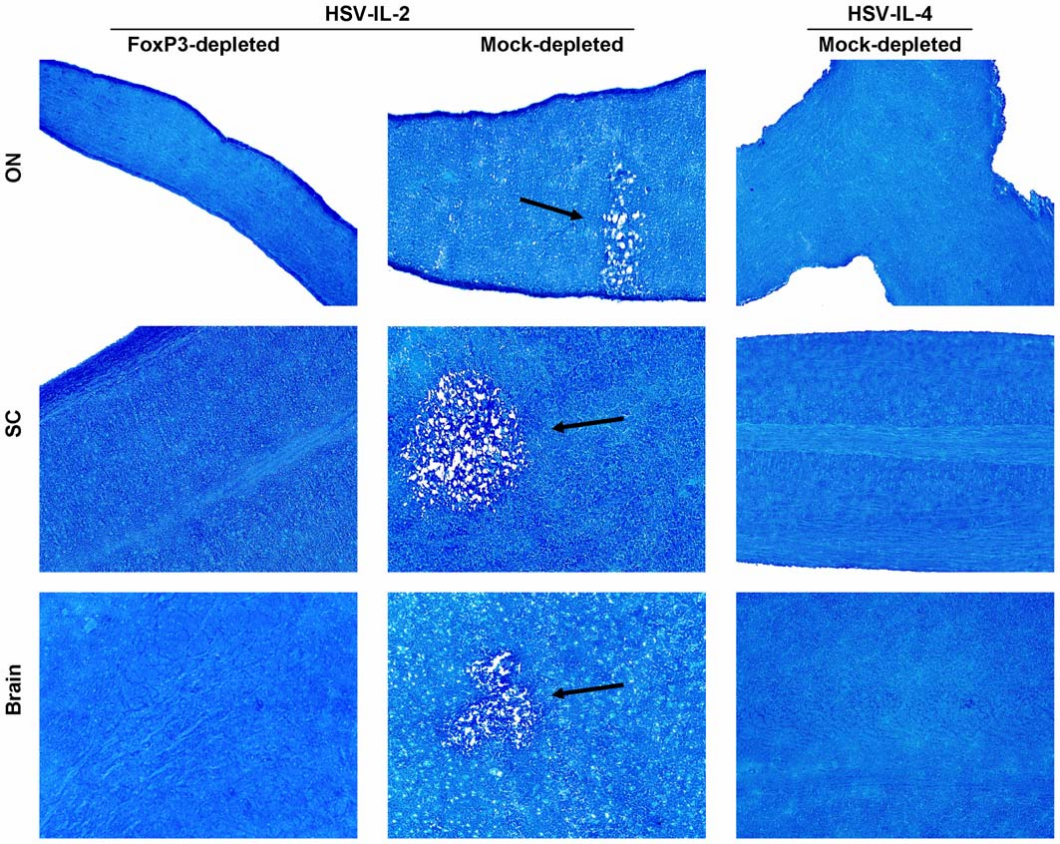
Demyelination in FoxP3^DTR^-depleted mice. FoxP3^DTR^ mice were infected ocularly with HSV-IL-2 or HSV-IL-4. Seventy-two and 24 hrs prior to infection, and 1, 3, 5, 7, and 9 days PI mice were depleted of their FoxP3 using diphtheria toxin as described previously [69]. After 14 days, ON, SC and brain were removed, sectioned, and stained for LFB. Arrows indicate areas of demyelination in ON, SC, and brains of HSV-IL-2 infected mice.

Our qRT-PCR analyses described above (Fig. 5) suggested that all transferred T cells irrespective of presence or absence of CD25 had similar levels of FoxP3 transcript in the brain of recipient SCID mice suggesting that FoxP3 may play a role in HSV-IL-2-induced CNS demyelination. To determine whether FoxP3 contributed to the HSV-IL-2-induced demyelination, FoxP3^DTR^ mice were depleted of FoxP3 using diphtheria toxin and infected ocularly with HSV-IL-2 or control HSV-IL-4. Demyelination was not detectable in ON, SC or brain of FoxP3 depleted mice infected ocularly with HSV-IL-2 (Fig. 6, Left Panels). However, demyelination was detected in CNS of mice that were mock-depleted and infected with HSV-IL-2 (Fig. 6, Middle Panels). As expected mice of that were mock-depleted and infected with HSV-IL-4 rather than HSV-IL-2 showed no sign of demyelination in ON, brain, or SC (Fig. 6, Right Panels). These results suggested that, FoxP3-positive T cells contribute to the HSV-IL-2-induced demyelination.

### Effect of endogenous IL-2 on the outcome of HSV-IL-2 infection

**Figure 7:**
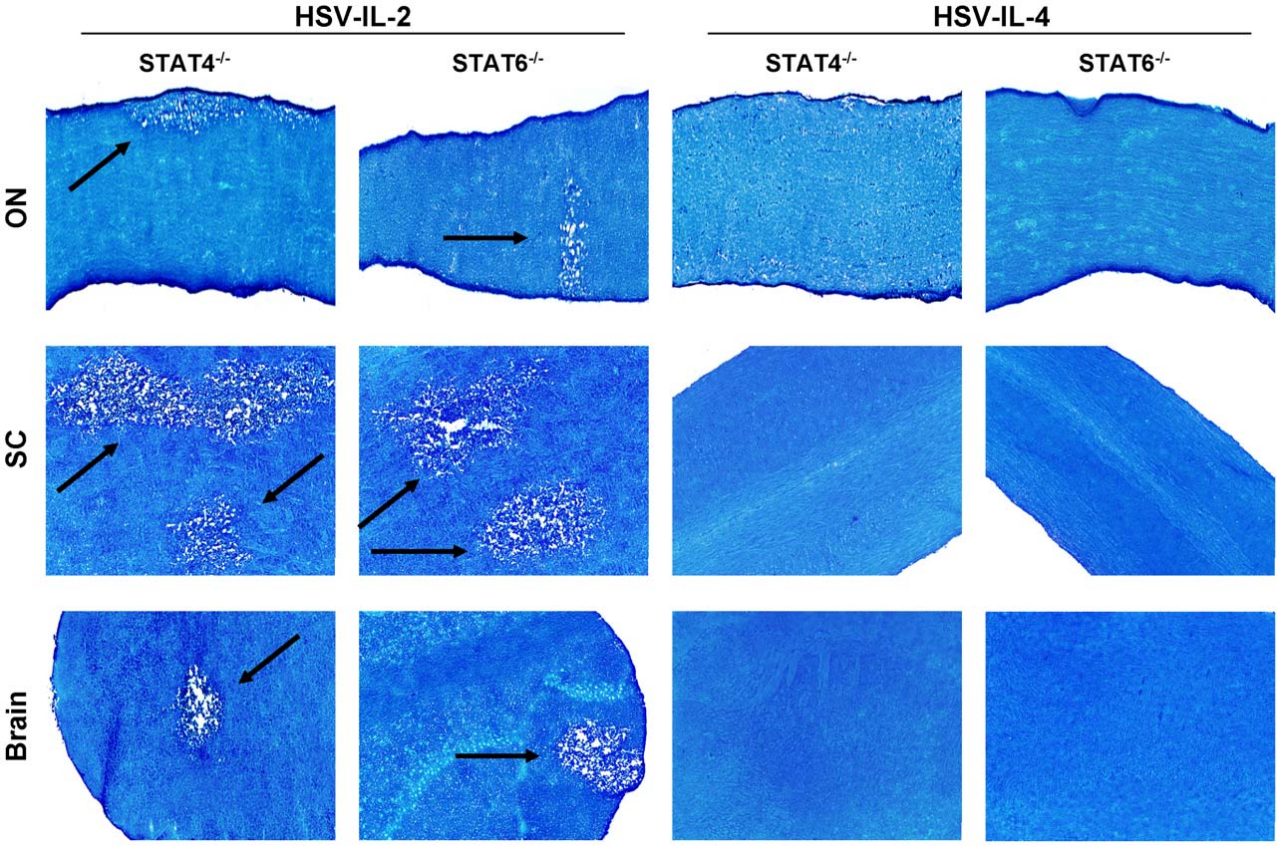
Demyelination in STAT4^−/−^ and STAT6^−/−^ mice. Female STAT4^−/−^ and STAT6^−/−^ mice were infected ocularly with HSV-IL-2 or HSV-IL-4 as described in Materials and Methods. After 14 days, ON, SC, and brains were removed, sectioned, and stained for LFB. Arrows indicate areas of demyelination in ON, SC, and brains of HSV-IL-2 infected mice.

Previously we have shown that splenocytes from mice infected with HSV-IL-2 induced a T_H_0/T_C_0 type of immune response during early phase of infection suggesting that the host IL-2 may contribute to demyelination [13], [17]. Moreover, our confocal microscopic analyses of double-stained brain sections from HSV-IL-2-infected mice suggested the presence of both exogenous IL-2 (produced by HSV-IL-2) and endogenous IL-2 (produced by host) [17]. To assess the possible involvement of host IL-2 in the HSV-IL-2-induced demyelination, we used BALB/c-STAT4^−/−^ mice, which do not mount a T_H_1 response, and BALB/c-STAT6^−/−^ mice, which do not mount a T_H_2 response. Mice were infected ocularly with 2×10^5^ PFU/eye of HSV-IL-2 or HSV-IL-4, sacrificed at day 14 PI and the ON, brain, and SC dissected and stained with LFB. Control wt BALB/c mice were infected similarly with HSV-IL-2 or HSV-IL-4. Representative photomicrographs of are shown in Figure 7. Demyelination was observed in the ON, SC, and brain of both STAT4^−/−^ and STAT6^−/−^ mice (Fig. 7, Left Panels, arrows). No such lesions or other signs of demyelination were observed in the ON, SC, and brain of either STAT4^−/−^ or STAT6^−/−^ mice infected with HSV-IL-4 (Fig. 7, Right Panels). Overall, more demyelination was detected in the SC of the STAT4^−/−^ and STAT6^−/−^ mice infected with HSV-IL-2 than in their brain or ON (Fig. 6, HSV-IL-2, Left panels). Similar results were obtained upon infection of wt BALB/c mice (data not shown). Thus, these results suggest that endogenously produced T_H_1 and T_H_2 cytokines did not contribute to CNS demyelination.

### Role of CD25 (IL-2rα) in HSV-IL-2-induced demyelination

**Figure 8:**
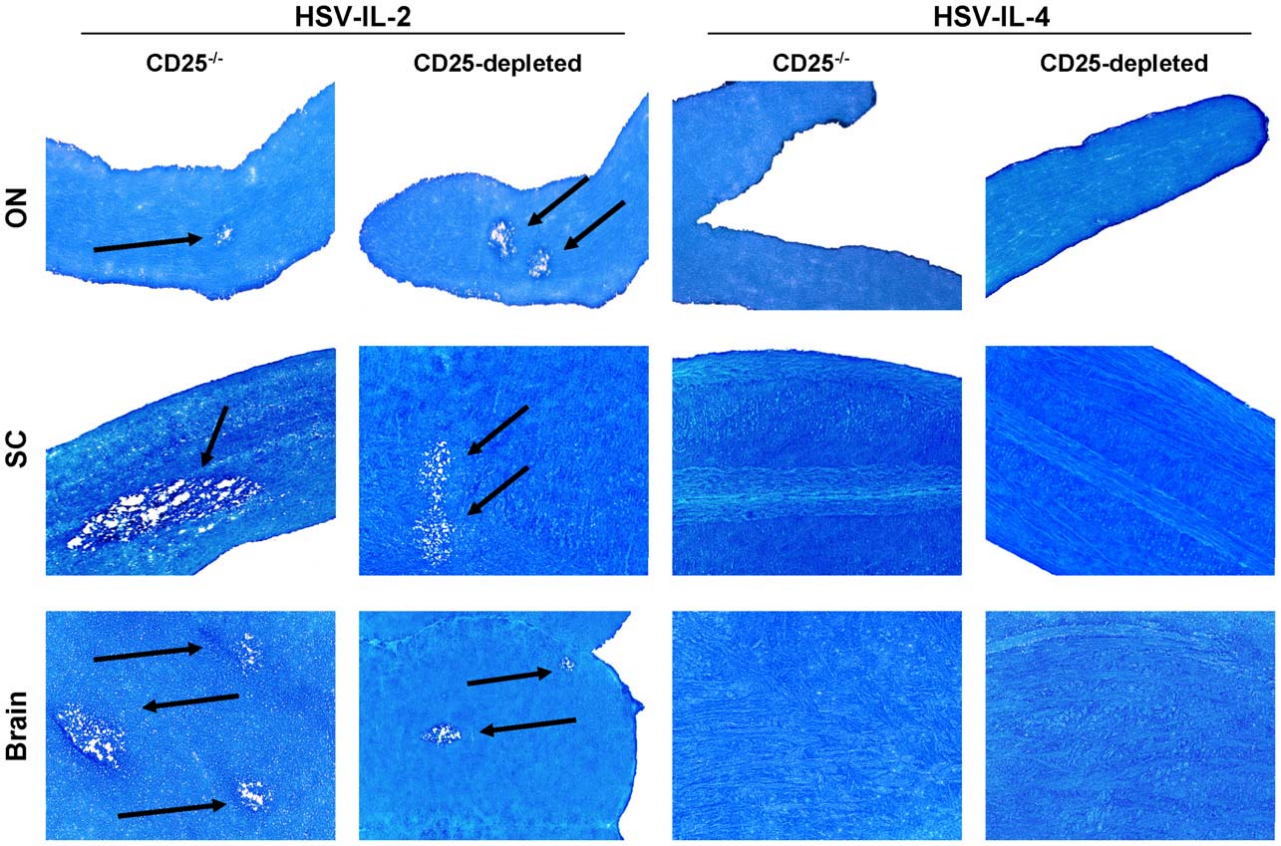
Role of CD25 (IL-2rα) in CNS demyelination. Female C57BL/6 IL-2rα^−/−^ mice were infected ocularly with HSV-IL-2 or HSV-IL-4 as described in Materials and Methods. For CD25 depletion, female C57BL/6 mice were infected ocularly with HSV-IL-2 or HSV-IL-4. Forty-eight hr prior to infection, and 1, 5, and 9 days PI mice were depleted of CD25 population as described in Materials and Methods. After 14 days, ON, SC, and brains were removed, sectioned, and stained for LFB. Arrows indicate areas of demyelination.

CD25, also known as IL-2rα, is a component of the high-affinity IL-2R, which increases the sensitivity of the receptor for IL-2 by more than 100-fold [24], [25]. Our transfer studies suggest that CD25 does not play any role in the HSV-IL-2-induced demyelination. To further determine the lack of CD25 involvement in HSV-IL-2-induced demyelination, we used C57BL/6-IL-2rα^−/−^ mice as well as depletion of CD25 in C57BL/6 mice using anti-CD25 mAb. Knockout mice and depleted mice were infected ocularly with HSV-IL-2 or HSV-IL-4. Fourteen days after infection, the mice were sacrificed and the ON, SC, and brain removed, post-fixed and stained with LFB. Representative photomicrographs of ON, SC, and brain sections from the knockout and depleted mice infected with HSV-IL-2 or HSV-IL-4 are shown in Figure 8. HSV-IL-2 virus induced demyelination in ON, SC, and brain of both CD25^−/−^ and CD25-depleted mice (Fig. 8, Left Panels), while mice infected with HSV-IL-4 did not show any sign of demyelination in their ON, SC, or brain (Fig. 8, Right Panels). Overall, the number of plaques detected in ON, SC, or brain of both knockout and depleted mice was similar to that of wt mice infected with HSV-IL-2 (not shown). These results were consistent with the results obtained in the adoptive transfer studies and confirm that HSV-IL-2-induced demyelination can occur independently of CD25 (IL-2rα).

### HSV-IL-2 suppresses IL-12p35 and IL-12p40 transcripts in BM-derived macrophages

**Figure 9:**
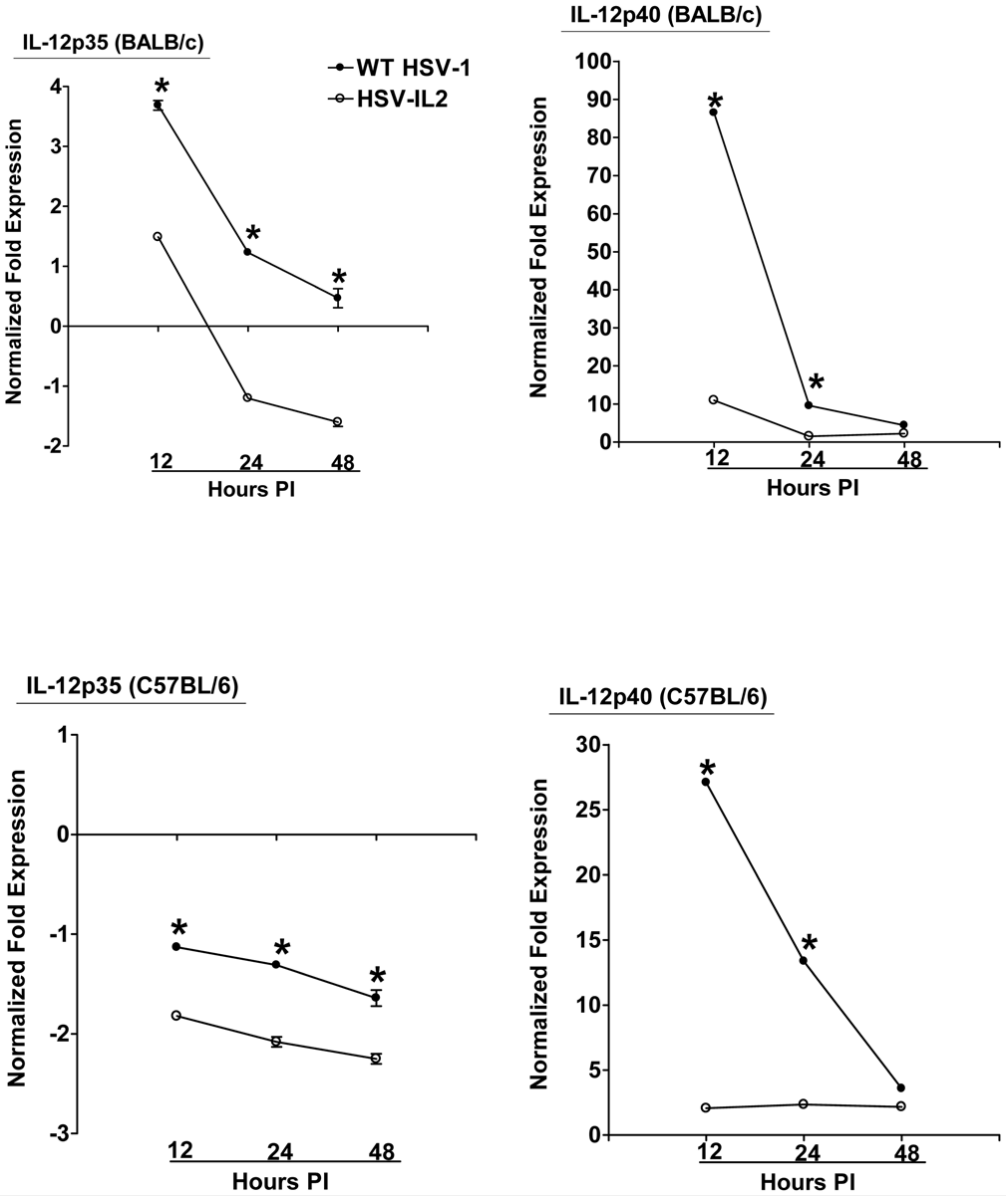
Level of IL-12p35 and IL-12p40 transcripts in macrophages infected with HSV-IL-2. Subconfluent monolayers of macrophages from BALB/c and C57BL/6 mice were infected with 10 PFU/cell of HSV-IL-2 or WT HSV-1. Total RNA was isolated 12, 24, and 48hr PI and TaqMan qRT-PCR was performed using IL-12p35- and IL-12p40-specific primers as described in Materials and Methods. IL-12p35 and IL-12p40 mRNA levels were normalized in comparison to each transcript in mock-infected macrophages. GAPDH was used as internal control. Each point represents the mean ± SEM (n = 6) from two separate experiments.

As we have detected an exacerbation of CNS demyelination in macrophage-depleted mice following ocular infection of mice with wt HSV-1 (unpublished results), we reasoned that the HSV-IL-2 induced demyelination could be due to suppression of the macrophages and an associated alteration in the expression of IL-12p35 and IL-12p40 transcripts. To test this possibility, we isolated macrophages from BALB/c and C57BL/6 mice and infected them with 10 PFU/cell of HSV-IL-2 or wt HSV-1, or mock infected them as we described previously [26]. Previously, we have shown that macrophages are infected with HSV-1 but the virus does not replicate in the infected cells [26]. The infected or mock-infected macrophages were harvested 12, 24, and 48 h PI, the total RNA was isolated, and IL-12p35 and IL-12p40 mRNAs levels were quantified by qRT-PCR. We performed TaqMan qRT-PCR on isolated RNA to determine the amount of IL-12p35 and IL-12p40 mRNAs in infected macrophages relative to levels of each transcript in the mock-infected macrophages. Cellular GAPDH mRNA was used as an internal control. Our results suggest that between 12 h and 48 h PI, the levels of IL-12p35 (Fig. 9, BALB/c) and IL-12p40 (Fig. 9, BALB/c) transcripts in the HSV-IL-2 infected macrophages was significantly lower than the levels of these transcripts in the wt HSV-1-infected macrophages. Similar results were observed in macrophages isolated from C57BL/6 mice (Fig. 9, C57BL/6). These results suggest that HSV-IL-2 infection alters the ratio of IL-12p35 and IL-12p40 transcripts in infected macrophages and that this could be a contributing factor in the development of the T-cell autoimmunity in the infected mice.

### Demyelination in HSV-IL-2-infected mice can be blocked by injection of IL-12p70 DNA

**Figure 10:**
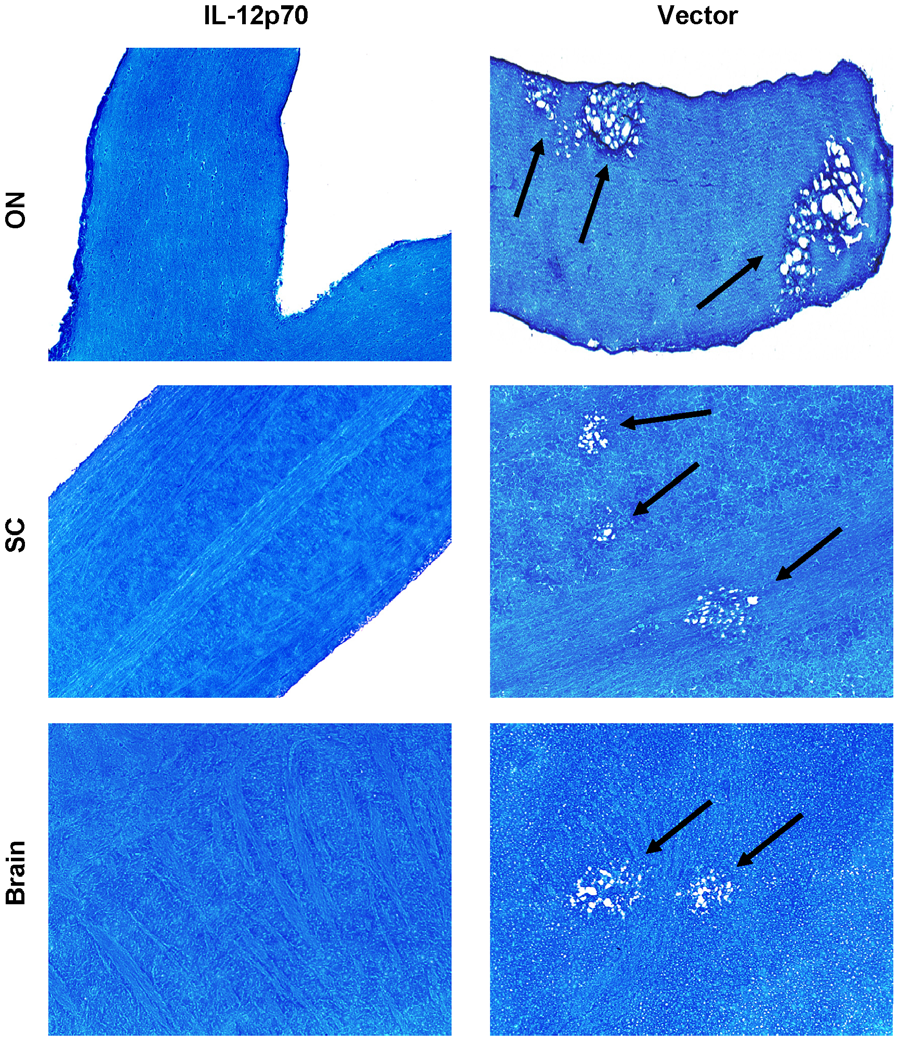
Blocking demyelination in HSV-IL-2 infected mice by IL-12p70 DNA injection. BALB/c mice were injected IM with IL-12p70 DNA or vector DNA as described in Materials and Methods. Four hours after the third DNA injected, mice were infected ocularly with HSV-IL-2. Representative ON, SC and brain sections on day 14 PI from infected mice are shown. The arrows indicate areas of demyelination.

Our results described above in Figure 9 suggested that HSV-IL-2 induced demyelination may be associated with an imbalance of IL-12p70. Thus, the absence of IL-12 function of macrophages may be the main contributing factor to HSV-IL-2- induced demyelination and the IL-12p70 arm of macrophage responses may be essential for prevention of demyelination. To confirm this hypothesis, we looked at the possibility of whether IL-12p70 injection in HSV-IL-2 infected mice may compensate for the imbalance of IL-12p35 and IL-12p40 transcripts and thus prevent demyelination in infected mice. BALB/c mice were immunized with IL-12p70 DNA or vector DNA and infected 4hr later with HSV-IL-2. Demyelination in ON, SC, and brain of infected mice was measured on day 14 PI. Demyelination was not observed in the ON, SC, and brain of BALB/c mice injected with IL-12p70 DNA (Fig. 10, IL-12p70). However, mice injected with vector DNA, displayed demyelination in their ON, SC, and brain (Fig. 10, Vector). Similar result was observed in C57BL/6 mice injected with IL-12p70 DNA and infected with HSV-IL-2 (not shown). However, demyelination was not blocked when mice were injected with IL-23, IL-27, or IL-35 and infected with HSV-IL-2 (not shown). Thus, our results suggest that demyelination in the CNS of the HSV-IL-2 infected mice is due to the downregulation of IL-12p70 expression by the macrophages.

## Discussion

**Figure 11:**
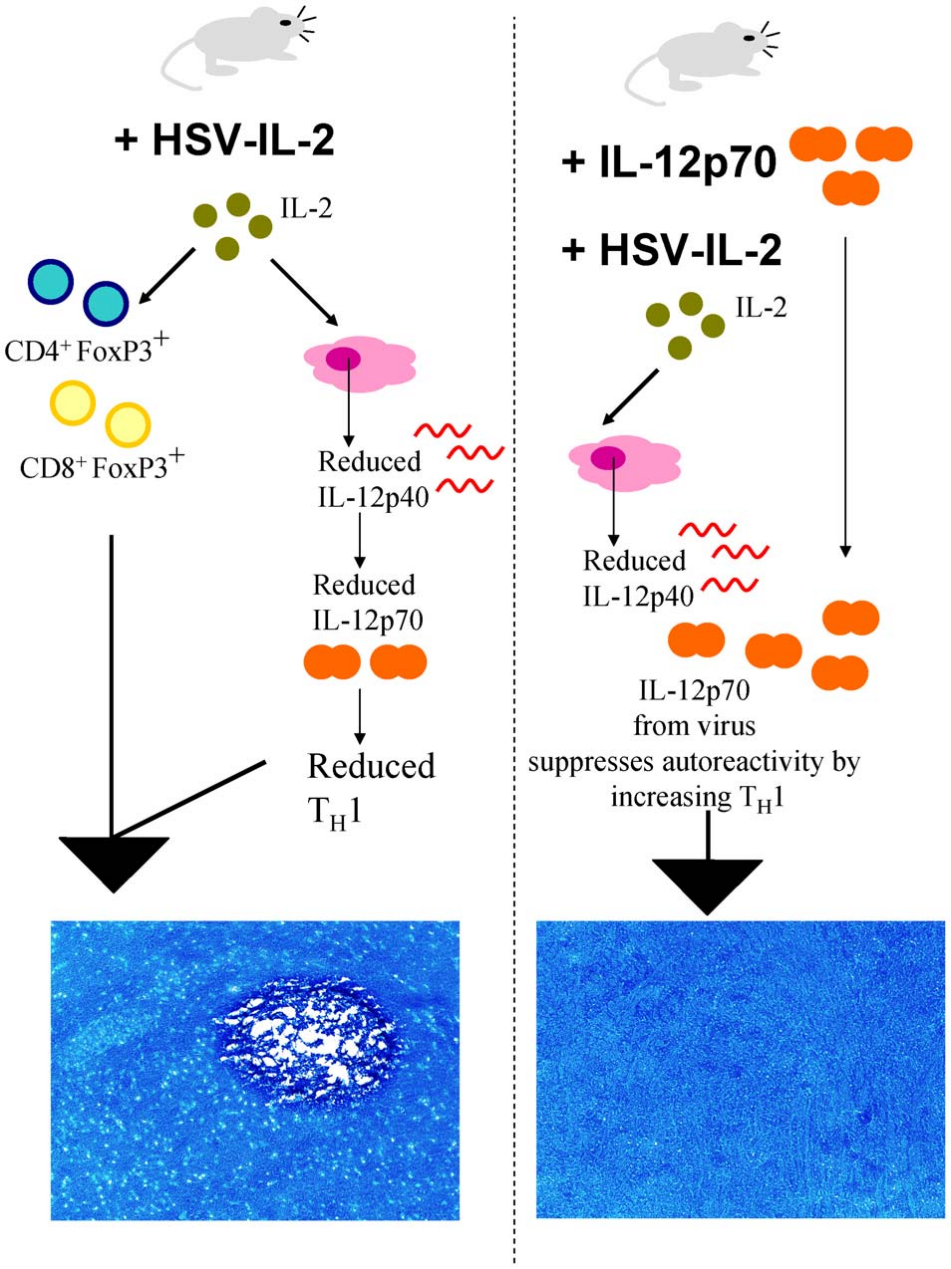
Proposed mechanism for HSV-IL-2 induced CNS demyelination. The cartoon demonstrates that when mice are infected with HSV-IL-2 they develop CNS demyelination but when they are co-infected with HSV-IL-2 and IL-12p70 this demyelination is blocked. We propose that this is caused via the effect of IL-2 on reducing the expression of IL-12p40 in macrophages. Less IL-12p40 means less of the heterodimer IL-12p70 leads to imbalance T_H_1 response and favors the production of more autoreactive CD4^+^FoxP3^+^ and CD8^+^ FoxP3^+^ T cells. Constitutive expression of IL-2 by HSV-IL-2 sustains an active autoreactive CD4^+^FoxP3^+^ and CD8^+^ FoxP3^+^ populations. The combination of FoxP3^+^ T cells and reduced T_H_1 leads to CNS demyelination (left side). In contrast, co-infection of IL-12p70 with HSV-IL2 compensates for the reduction in IL-12p70 caused by IL-2 leading to functional T_H_1 populations and reduced demyelination (right side).

In order to mimic the elevation of IL-2 expression that is typical of MS we have used the neurotropic potential of HSV-1 and the unique characteristics of the *LAT* (latency-associated transcript) promoter that is active in most cell types to extend expression of murine IL-2 [13]. This model of MS in which mice are infected with HSV-IL-2 differs from most animal models of MS that are based on either the autoimmune model [27] or the viral model [21] in that this model incorporates both viral and immune aspects of the disease process. Recently, we reported that this recombinant virus causes CNS demyelination in four different strains of mice and that the demyelination is more severe in female then male mice [18]. A summary of the results obtained here with regards to the mechanism of HSV-IL-2-induced CNS demyelination and blocking CNS demyelination is presented schematically in Figure 11. The results of the present study, in which we used both the BALB/c and C57BL/6 mouse strains, indicate that B-cells, DCs, and NK cells do not play a role in the HSV-IL-2-induced demyelination. In contrast, evidence for involvement of both CD4^+^ and CD8^+^ T cells in the HSV-IL-2-induced demyelination was observed using knockout mice, depletion studies and transfer studies. Moreover, we show that the CD8^+^ T cells played a more significant role in HSV-IL-2 induced demyelination than the CD4^+^ T cells. These findings are consistent with the published data concerning histologic analyses of specimens obtained from patients with MS at autopsy, which have shown a possible correlation between the presence of CD4^+^ and CD8^+^ T cells and the development of demyelinating lesions [19], [20]. The results are also consistent with the reports that demyelination induced by mouse hepatitis virus (MHV) is associated with both T cell types [21]. In the EAE model of MS, it was believed originally that only CD4^+^ T cells were involved in the CNS demyelination [28], but later studies showed that CD8^+^ T cells can also induce demyelination [19].

We extended the studies to evaluate the role of “naïve” and HSV-IL-2 “effector” T cells in CNS demyelination using SCID mice. Based on their constitutive expression of CD25 (the IL-2rα-chain), T cells can be divided into CD25^+^ and CD25^−^ subpopulations [29]. Although it has been reported that only CD25^+^ T cells constitutively express forkhead/winged-helix transcription factor (Foxp3), recent studies have shown that CD25^−^ T cells also can express FoxP3 [22], [30], [31]. In the present study, we show that both CD4^+^ and CD8^+^ T cells irrespective of their CD25 status express the FoxP3 gene at equal levels. In line with the detection of FoxP3 transcript in brain of recipient SCID mice, our FoxP3 depletion studies suggest that FoxP3 is contributing to CNS demyelination. Our results suggest that T cells, whether they express CD25 or not, contribute to the development of autoimmunity in this model and this could be due to the fact that both the CD25^+^ and CD25^−^ cells express similar levels of FoxP3. FoxP3 expression on Teff T cells is transient and its expression in Teff cells is not enough to convert them into Treg, although under repetitive *in vitro* stimulation this could occur [32]. FoxP3 expression in Teff cells and its maintenance in Treg cells are both dependent on IL-2 [33], [34]. Consequently, in the presence of IL-2-expressing HSV-1 T cells irrespective of being regulatory or effector cells all expressing FoxP3 and HSV-IL-2-induced CNS demyelination is dependent on FoxP3. Thus, our result is similar to the results of previous studies that suggest that both CD8^+^ Treg [35], [36], [37] and CD4^+^ Treg [38], [39], [40], [41], [42] can cause autoimmunity.

In addition to T cells, macrophages have been implicated in CNS pathology and MS [43], [44]; however, they may play only an indirect role. The macrophage plays a variety of roles in the immune defense system, including phagocytosis, tumor cytotoxicity, cytokine secretion and antigen presentation [45], [46], as well as cross-presentation of antigens to naive T cells *in vivo* [47], [48], [49]. A number of factors are known to “activate” or engage macrophages in these activities, including viral infection. In this study, we show that HSV-IL-2 infection of macrophages alters the balance of IL-12p35 and IL12p40 transcripts, which theoretically could favor the development of autoreactive T cell in the HSV-IL-2 infected mice. This possibility is supported by our results that showed that injection of mice with IL-12p70 DNA blocked HSV-IL-2-induced demyelination. Similar results were obtained when HSV-IL-2 infected mice were also infected with a recombinant HSV-1 expressing IL-12p70 (not shown). Co-infection of mice with HSV-IL-2 + HSV-IL-12p35, HSV-IL-2 + IL-12p40 alone, or a mixture did not block demyelination (not shown). Similarly injection of IL-12p35 DNA alone, IL-12p40 DNA alone, or a mixture did not block demyelination (not shown). Thus, the in vivo biological activity of IL-12p70 is dependent on the use of a heterodimer. Macrophages are the main source of IL-12 production [50], [51]. IL-12 was considered to be a critical cytokine in the pathogenesis of EAE [52] and later studies showed that although the IL-12p40 component of IL-12 is involved in EAE-induced CNS pathology this effect is mediated by the binding of the IL-12p40 to IL-23p19 rather than its binding to IL-12p35 [53], [54]. Thus, the imbalance of IL-12p40 and IL-12p35 in the HSV-IL-2 infected mice may be responsible for a shift in T_H_1 and T_H_2 pattern of cytokine responses as we reported previously [17], and this shift could be responsible for the development of the autoreactive T cells. This would be consistent with our detection of demyelination in both STAT4^−/−^ (no T_H_1 response) and STAT6^−/−^ (no T_H_2 response) mice infected with HSV-IL-2 virus. Previously, it was shown that STAT6-deficient mice develop a more severe clinical course of EAE as compared with wild-type or STAT4 knockout mice [55], while it was reported that IL-2-knockout mice developed less demyelination compare with their wt counterparts [12]. Thus, some studies suggest that T_H_1 cells are protective while other studies suggest they are pathogenic. These discrepancies could be due to the use of different antigen, mouse strain, or the methods of measurement of autoreactivity.

Previously it was shown that IL-2 was essential for the survival of T regs *in vivo* [56], [57], and exogenous administration of IL-2 can boost antigen-specific T cell responses and delay the death of superantigen-reactive T cells [58], [59], [60]. Furthermore, IL-2/IL-2 mAb complexes also was shown to increase biological activity of preexisting IL-2 leading to expansion of CD8^+^ T cells as well as CD4^+^ T regs in vivo [61]. Our results suggest that constitutive expression of IL-2 by HSV-IL-2 may prolong the survival of autoreactive T cells in addition to enhancing the homing of activated T cells to the CNS. Previously it was shown that nTreg cells suppress IL-2 mRNA transcription and proliferation of CD4^+^ and CD8^+^ Teff cells [62]. This could be the reason that in the present study we did not detect CNS demyelination in SCID mice that received nCD4^+^CD25^+^ T cells following ocular infection with HSV-IL-2. In MS a functional defect of nTreg cells has been reported [63], despite a frequency of nTrg cells that is similar in patients and in healthy individuals [64]. Similarly to MS, a normal number of nTreg cells but with decreased function had been described in type I diabetes (TID) [65] and in autoimmune polyglandular syndrome type II [66]. In contrast to CD4^+^ T cell transfer, both naive and effector CD8^+^ T cells caused CNS demyelination in HSV-IL-2 infected mice, whereas in KOS-infected mice only effector CD8^+^ T cells caused demyelination. This is similar to the results of previous studies that suggest that CD8^+^ Treg cells can cause autoimmunity [35], [36], [37].

In addition to the expression of the IL-2 increasing T cell survival, we find that it also causes an alteration in the IL-12p70 production by macrophages. This alteration in the IL-12p70 component of the macrophage response and increase in T cell survival in the presence of IL-2 may lead to the development of autoimmune T cells. In line with the inhibitory effects of HSV-IL-2 on IL-12p70 function of macrophages, we have shown that depletion of macrophages also causes CNS demyelination following ocular infection of depleted mice by wild-type HSV-1. Similar to this study, demyelination in macrophage depleted mice can be blocked by IL-12p70 DNA injection (manuscript in preparation). Thus, our results suggest that communication between macrophages and T cells *via* the production of IL-12p70 by the macrophages acts as a critical suppressor of T-cell autoreactivity. This inter-relationship between the macrophages and T cells can be affected by elevated expression of IL-2, which leads to loss of the suppressive effect; however, the inhibitory effects of the IL-2 can be restored by IL-12p70 supplementation.

## Materials and Methods

### Ethics Statement

All animal care and experimental protocols were conducted in accordance with the regulations of the institutional care and use committee at the Cedars-Sinai Medical center and the NIH *Guide for the Care and Use of Laboratory Animals* (ISBN 0-309-05377-3).

### Mice, viruses, and cells

Female BALB/c, BALB/c-STAT4^−/−^, BALB/c-STAT6^−/−^, BALB/c-CD19^−/−^ (B cell-deficient), BALB/c-SCID, C57BL/6, C57BL/6-CD4^−/−^, C57BL/6-CD8^−/−^, and C57BL/6-IL-12rα^−/−^ (CD25-deficient) mice 6-weeks of age were purchased from the Jackson Laboratory (Bar Harbor, ME). Female CD19^−/−^ (B cell-deficient) and hemizygous C.FVB-Tg (Itgax-DTR/GFP) 57Lan/J mice on a BALB/c background were obtained from The Jackson Laboratory, while FoxP3^DTR^ mice in C57BL/6 background were a gift from Alexander Y Rudensky. CD19^−/−^, C.FVB-Tg (Itgax-DTR/GFP) 57Lan/J, and FoxP3^DTR^ mice were bred at Cedars-Sinai Medical Center.

Six-week-old female BALB/c or C57BL/6 (The Jackson Laboratory) mice were used as a source of bone marrow (BM) cells that were used to generate macrophages in culture as we described previously [26]. Briefly, BM cells were collected by flushing the femurs with PBS. The cells were pelleted and resuspended briefly in water to lyse red blood cells then stabilized by adding complete medium (RPMI 1640, 10% fetal bovine serum, 100 U/ml penicillin, 100 µg/ml streptomycin, 2 mM L-glutamine). After centrifugation and resuspension in complete medium supplemented with macrophage-colony stimulator factor (M-CSF) (100 ng/ml; Peprotech, NJ), the cells were plated in non-tissue culture plastic Petri dishes (cells from 1 bone per 10 cm dish) and incubated for 5 days at 37°C with CO_2_. After 5 days, the media was removed and the adherent cells were recovered by incubating the cells with Versene (Invitrogen, San Diego, CA) for 5 min. at 37°C. The cells were washed, counted, and plated onto tissue-culture dishes for use the following day.

Plaque-purified HSV-1 strains, McKrae (wild type) or KOS and HSV-1 recombinant viruses expressing IL-2 and IL-4 (HSV-IL-2, HSV-IL-4) were grown in rabbit skin (RS) cell monolayers in minimal essential medium (MEM) containing 5% fetal calf serum (FCS), as described previously [13], [14], [16]. McKrae virus is virulent at an infectious dose of 2×10^5^ plaque forming units (PFU)/eye, whereas the KOS, HSV-IL-2, HSV-IL12p70 (M002), and HSV-IL-4 viruses are attenuated. Previously we have shown that the recombinant HSV-IL-2 is expressing IL-2 at high levels in different tissues [13].

### Ocular infection

Mice were infected ocularly with 2×10^5^ PFU of McKrae, KOS, recombinant HSV-IL-2, or HSV-IL-4 per eye. Each virus was suspended in 5 µl of tissue culture media and administered as an eye drop. In contrast to mice with the C57BL/6 background that are refractory to McKrae infection, mice with the BALB/c background are highly susceptible to McKrae infection. Thus, mice with BALB/c background were infected with KOS rather then McKrae virus. Corneal scarification was not used.

### DNA immunization

The complete open-reading frame (ORF) for IL-12p70 (pORF-mIL12) was purchased from InvivoGen (San Diego, CA). Plasmid DNA was purified using cesium chloride gradient. In each experiment, five mice per group were injected intramuscularly (IM) (into each of the quadriceps) using a 27 gauge needle with 100 µg of cesium chloride-purified DNA in a total volume of 50 µl of PBS 3 times. DNA injections were done 14 days, 7 days, and 4 hrs before ocular infection. As a negative control, we used mock-treated vaccinated mice that were similarly injected with vector DNA alone.

### Depletion of dendritic cells (DCs) and FOxP3

Female BALB/c-DTR mice were depleted of their DCs by treatment with 100 ng of diphtheria toxin (DT), which was administered in 100 µl of PBS and injected intraperitoneally as we described previously [67], [68]. Briefly, the mice were administered DT 24 h before ocular infection, followed by four additional treatments on days +1, +4, +7, and +10 post infection (PI). FoxP3^DTR^ mice were depleted of their FoxP3 by treatment with DT as described previously [69]. Briefly, the mice were administered DT 72 and 24 h before ocular infection, followed by 5 additional treatments on days +1, +3, +5, +7, and +9 PI. This regimen of treatments reduced each population by more than 97% as confirmed by FACS analysis of spleen cells 24 hours after the second depletion as described previously [69].

### Depletion of CD4^+^ and CD8^+^ T cells, and CD25^+^ cells

Each mouse received an intraperitoneal injection of 100 µg of purified GK1.5 (anti-CD4**^+^**), or 2.43 (anti-CD8**^+^**), or both GK1.5 and 2.43, or PC61.5.3 (anti-CD25) monoclonal antibodies (NCCC, Minneapolis, MN) in 100 µl of PBS, -5 and -2 days before ocular infection. The injections were then repeated on days +1, +4, +7, and +10 relative to ocular infection. Control mice were depleted with an irrelevant mAb of the same isotype. The efficiency of CD4**^+^**, CD8**^+^**, and CD25^+^ depletion was monitored by FACS analysis of splenocytes 24 h after the second depletion and before ocular infection. After the second depletion, more than 95% of CD4+ T cells, CD8+ T cells, or CD25+ T cells were depleted from the spleens as we described previously [72]. However, based on our results these residual of T cells did not have any effect on inducing demyelination when both CD4+ and CD8+ T cells were depleted.

### Depletion of NK cells with anti-asialo GM1

One mg of rabbit anti-asialo GM_1_ antibody (Wako Chemicals, Dallas, TX) was dissolved in 1 ml of PBS and each mouse received multiple intraperitoneal injections of 100 µg of antibody in 100 µl of PBS. The first depletion was done -5 days before ocular infection and this was followed by five additional depletions on days −2, +1, +4, +7, and +10 PI as we described previously [70]. After the second depletion, more than 93% of NK cells were depleted from the spleens as we described previously [70]. Control mice were treated with an equal concentration of freeze-dried normal rabbit serum in PBS.

### Preparation of ON, SC, and brain for pathologic analysis

The ON, SC, and brain of infected mice were removed at necropsy on day 14 PI. ON, SC, and brain were collected from experimental and control mice, then placed in Tissue-TeK OCT embedding medium (SaKura Fintek, Torrence, CA) and then stored at –80°C. Transverse sections of each tissue, 8-10 µm thick, were cut, air-dried overnight, and fixed in acetone for 3 min at 25°C [71]. Demyelination in each section was confirmed by monitoring adjacent sections.

### Analysis of demyelination using Luxol Fast Blue (LFB) staining

The presence or absence of demyelination in ON, SC, and brain of infected mice was evaluated using LFB staining of formalin-fixed sections of ON, SC, and brain as we described previously [17]. Every 4th section of ON, SC, and brain was stained with LFB.

### Adoptive transfer of T cells

Donor BALB/c mice that were either mock-infected or ocularly infected with HSV-IL-2 were sacrificed on day 5 PI, the spleens were pooled, and single-cell suspensions prepared as described previously [72]. Naive T cells (*i.e.*, nCD4^+^CD25^+^, nCD4^+^CD25^−^, nCD8^+^CD25^+^, nCD8^+^CD25^−^) from mock-infected mice, and effector T cells from HSV-IL-2-infected mice (*i.e.*, effCD4^+^CD25^+^, effCD4^+^CD25^−^, effCD8^+^CD25^+^, effCD8^+^CD25^−^) were isolated using magnetic beads as described by the manufacturer (Miltenyi Biotec, Auburn, CA). Each recipient SCID mouse was injected once with 2×10^5^ cells in 300 µl of MEM intraperitoneally. The control mice received 300 µl MEM alone. The recipient and control mice were infected ocularly with KOS or HSV-IL-2 virus 4 h after transfer of the cells.

### Infection of BM-derived macrophages *in vitro*

Bone marrow (BM) for the generation of mouse macrophages in cultures were isolated by flushing femurs and tibiae with PBS as we described previously [26]. Monolayers of macrophages isolated from BALB/c or C57BL/6 mice were infected with 10 PFU/cell of HSV-1 strain KOS or HSV-IL-2 or mock-infected. One hour after infection at 37°C, virus was removed and the infected cells were washed three times with fresh media and fresh media was added to each well. The monolayers including media were harvested at 12, 24, and 48 h PI. RNA preparation was done as we described previously [26]. Briefly, frozen cells were resuspended in TRIzol and homogenized, followed by addition of chloroform, and subsequent precipitation using isopropanol. The RNA was then treated with DNase I to degrade any contaminating genomic DNA followed by clean-up using a Qiagen RNeasy column as described in the manufacturer's instructions. The RNA yield from all samples was determined by spectroscopy (NanoDrop ND-1000, NanoDrop Technologies, Inc., Wilmington, Delaware). Finally, 1000 ng of total RNA was reverse-transcribed using random hexamer primers and Murine Leukemia Virus (MuLV) Reverse Transcriptase from the High Capacity cDNA Reverse Transcription Kit (Applied Biosystems, Foster City, CA), in accordance with the manufacturer's recommendations.

### TaqMan Real-Time PCR (qRT-PCR)

The expression levels of IL-12p35 and IL-12p40 genes in BM-derived macrophages and expression of CD4, CD8, and FoxP3 in brain of recipient SCID mice were evaluated using commercially available TaqMan Gene Expression Assays (Applied Biosystems, Foster City, CA) with optimized primer and probe concentrations as we described previously [73], [74]. Cellular GAPDH gene expression was used as an internal control. Primer-probe sets consisted of two unlabeled PCR primers and the FAM^TM^ dye-labeled TaqMan MGB probe formulated into a single mixture. The primers and probe used were as follows: 1) IL-12p35 (ABI ASSAY I.D. Mm00434165_m1 – Amplicon length  = 68 bp); 2) IL-12p40 (ABI ASSAY I.D. Mm 01288992_m1 – Amplcon length  = 109 bp); 3) GAPDH (ABI ASSAY I.D. m999999.15_G1 - Amplicon Length  = 107 bp); and 4) FoxP3 (ABI ASSAY I.D. Mm00475164_m1 – Amplicon length  = 80bp).

Quantitative real-time PCR was performed as we described previously [73]. Real-time PCR was performed in triplicate for each sample from each time point. Relative gene expression levels were normalized to the expression of the GAPDH housekeeping gene (endogenous loading control).

### Statistical analysis

Fisher's exact tests were performed using the computer program Instat (GraphPad, San Diego) to compare demyelination in infected mice with the absence of demyelination in control groups. Results were considered statistically significant when the *P* value was <0.05.

## Supporting Information

Figure S1 **Severity of CNS demyelination in knockout and depleted mice infected with HSV-IL-2.** The entire brain, SC and ON of each of the 5 animals described in Figs. 2 and 3 were sectioned and every 4 slides of each tissues were stained. The numbers of demyelination plaques in the entire sections of ON, SC and brain were counted. Data are presented as percent of sections with plaques per total sections stained (number on each bar graph shows the number of section showing plaques/total stained section). Panels: A) Percent of plaque/section in C57BL/6-CD4^−/−^, C57BL/6-CD8^−/−^, and WT C57BL/6 mice; and B) Percent of plaque/section in CD4-depleted, CD8-depleted, both CD4- and CD8-depleted, and WT mock depleted C57BL/6 mice.
(TIF)
