# Supplementary figures and images for: IL-2 Suppression of IL-12p70 by a Recombinant HSV-1 Expressing IL-2 Induces T-Cell Auto-Reactivity and CNS Demyelination
Source: PLoS One. 2011 Feb 18;6(2):e16820. doi: 10.1371/journal.pone.0016820 (PMC3041759; doi:10.1371/journal.pone.0016820)

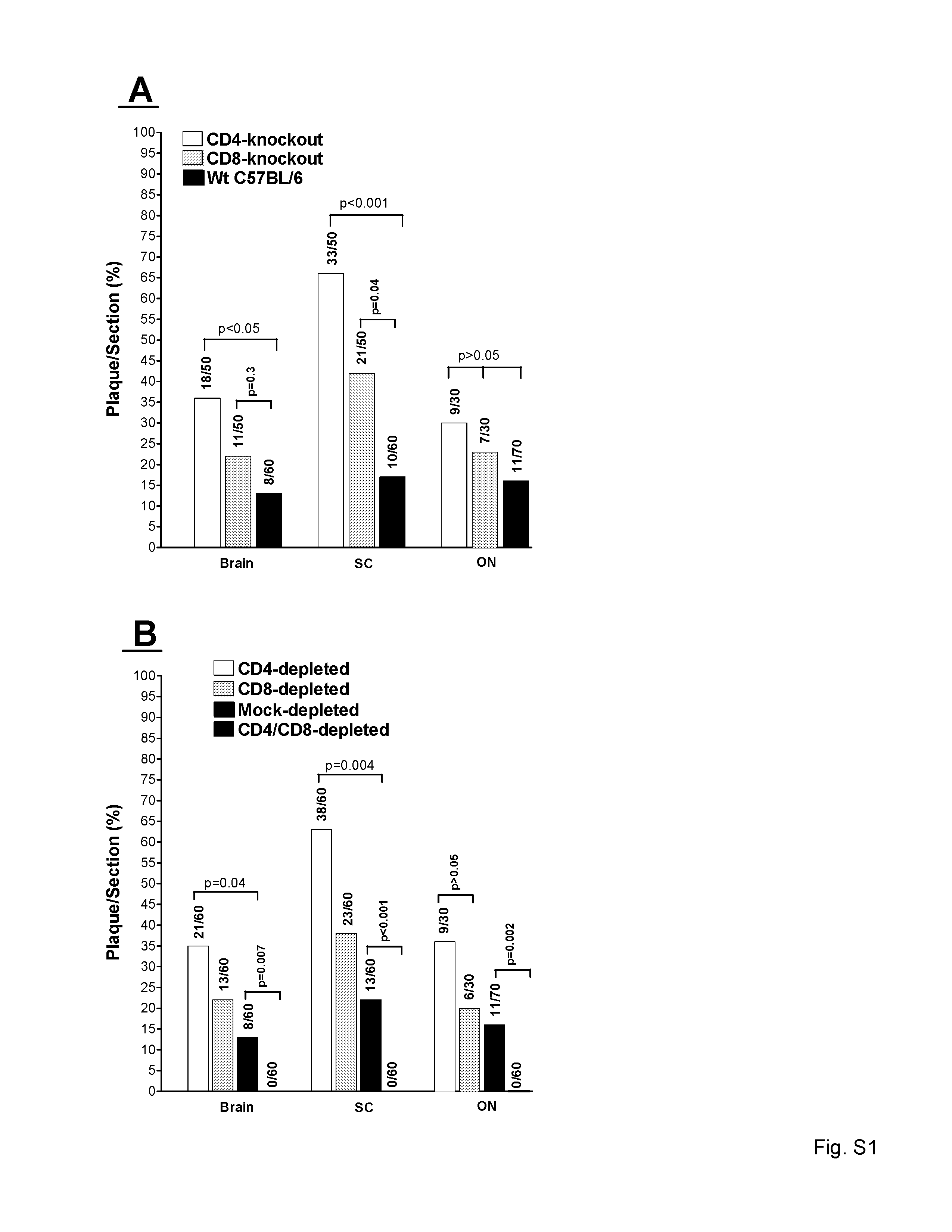

Supplement: Figure S1 — Severity of CNS demyelination in knockout and depleted mice infected with HSV-IL-2. The entire brain, SC and ON of each of the 5 animals described in Figs. 2 and 3 were sectioned and every 4 slides of each tissues were stained. The numbers of demyelination plaques in the entire sections of ON, SC and brain were counted. Data are presented as percent of sections with plaques per total sections stained (number on each bar graph shows the number of section showing plaques/total stained section). Panels: A) Percent of plaque/section in C57BL/6-CD4−/−, C57BL/6-CD8−/−, and WT C57BL/6 mice; and B) Percent of plaque/section in CD4-depleted, CD8-depleted, both CD4- and CD8-depleted, and WT mock depleted C57BL/6 mice. (TIF) [file pone.0016820.s001.tif]
